# Does COVID-19 vaccination protect against pulmonary embolism?

**DOI:** 10.1186/s44158-023-00097-4

**Published:** 2023-05-30

**Authors:** Simona Tantillo, Nicola Cilloni, Martina Guarnera, Francesco Talarico, Mario Citino, Mauro Silingardi, Leonardo Catalano, Michele Imbriani

**Affiliations:** 1 Department of Anaesthesia, Intensive Care and EMS, Ospedale Maggiore “Carlo Alberto Pizzardi”, Bologna, Italy; 2 Department of Internal Medicine, Ospedale Maggiore “Carlo Alberto Pizzardi”, Bologna, Italy; 3 Department of Diagnostic Imaging, Radiology Unit, Ospedle Maggiore “Carlo Alberto Pizzardi”, Bologna, Italy

**Keywords:** COVID-19, Pulmonary embolism, Vaccinated patients, Unvaccinated patients

## Abstract

The prevalence of venous thromboembolism (VTE) in COVID-19 patients is highly variable, depending on methodological and clinical factors, among which vaccination (1). The hypothesis of a possible protective role of vaccination in preventing pulmonary embolism (PE) in hospitalized COVID-19 patients has not been explored. The aim of the study was to evaluate PE prevalence in vaccinated versus unvaccinated hospitalized COVID-19 patients. We conducted a retrospective case–control study from 2021/11/01 to 2022/01/15; we reviewed all the chest computed topographies (chest-CT) performed because of a clinical suspicion for PE at our Institution. Sixty-two patients were included in the study: 27/62 (43.5%) were vaccinated and 35/62 (56.4%) were not. Vaccinated patients were older and with more comorbidities than unvaccinated people. Overall, PE was diagnosed in 19/62 patients (30.1% prevalence). CT Severity Score (CT-SS) differs between the two groups; not vaccinated patients had a more severe CT imaging than the vaccinated (< 0.00005). PE prevalence in ICU was 43.2% (16/37 patients), while in the Internal Medicine ward, it was 12% (3/25 cases). PE was significantly higher among unvaccinated people: 16/35 (45.7%) vs 3/27 (11.1%), OR *p* = 0.04. We observed a strong association between vaccination and protection from PE in hospitalized COVID-19 patients: morbidity was significantly lower in vaccinated versus not vaccinated patients. The issue of the protective role of vaccination in COVID-19-associated VTE should be addressed in adequately designed and powered future prospective studies.

## Introduction

The prevalence of venous thromboembolism (VTE) in patients with COVID-19 is highly variable, depending on methodological and clinical factors, among which vaccination [1]. The hypothesis of a possible protective role of vaccination in preventing pulmonary embolism (PE) in hospitalized COVID-19 patients has not been explored. A retrospective population-based study on ambulatory patients, showing a reduced VTE incidence in vaccinated patients at the 30-day follow-up, is the only evidence [2]. The aim of the study is to evaluate PE prevalence in vaccinated versus not-vaccinated hospitalized COVID-19 patients.

## Methods

From 2021/11/01 to 2022/01/15, we reviewed all the chest computed tomographies (chest-CT) performed because of a clinical suspicion (according to Well’s score) for PE in patients with COVID-19 pneumonia. Approval was obtained by the local ethic committee. All CT scans were reviewed by two expert radiologists (25 years of experience), who were blind to the vaccination status of patients. PE was classified as troncular, lobar, segmental, or sub-segmental, according to the location of the most proximal luminal defect. The severity of lung damage was classified according to CT Severity Score (CT-SS) [3]. Inclusion criteria: COVID-19 patients admitted in the Intensive Care Unit (ICU) or Internal Medicine. Exclusion criteria: history of VTE, thrombophilia, full anticoagulant therapy, active cancer, major trauma or major surgery in the last 30 days, bacterial sepsis. Primary endpoint: clinically overt, symptomatic PE diagnosed with chest-CT. Patients were managed according to current WHO recommendations (antithrombotic prophylaxis with sc enoxaparin 4000 U/die). Patients were considered vaccinated with at least 2 doses of Pfizer or 2 doses of Moderna or 1 of Johnson vaccine, evaluating the complete vaccination cycle 4 weeks after the administration of the last dose. Discrete variables were expressed as absolute values and percentages and continuous variables as median and interquartile range. Variables were compared using chi-square test (or Fisher’s exact test if needed) and *t*-tests as appropriate. Statistical significance was accepted if *P*-value was < 0.05.

## Results

**Fig. 1:**
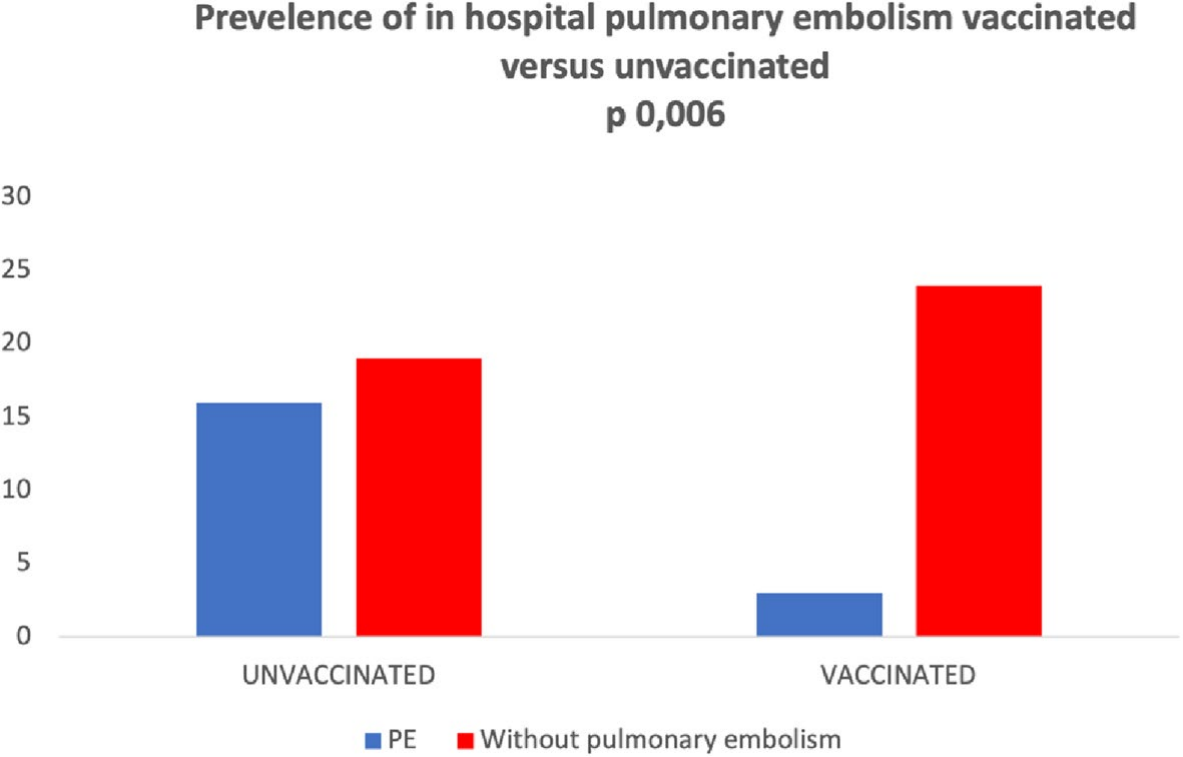
Comparison between vaccinate and unvaccinated COVID patients

Sixty-two patients were included in the study: 27/62 (43.5%) were vaccinated and 35/62 (56.5%) were not. The clinical characteristics are described in Table 1. Vaccinated patients were older and with more comorbidities than unvaccinated people. Overall, PE was diagnosed in 19/62 patients (30.6% prevalence). CT-SS differs between the two groups; unvaccinated patients had a more severe CT imaging (17.82 ± 4.87) than the vaccinated (10.81 ± 4.44) (*p* < 0.00005). PE prevalence in ICU was 39.4% (13/33 patients), while in Internal Medicine, it was 20.7% (6/29 cases). PE was significantly higher among unvaccinated people 16/35 (45.7%) vs 3/27 (11.1%), OR 6.73 (95% CI 1.7–26.5, *p* = 0.006) (Fig. 1). Thirteen cases were registered in ICU (12 in unvaccinated and 1 in vaccinated patients) and six cases occurred in the Medical Ward (2 in vaccinated and 4 in unvaccinated people).

**Table 1:** Clinical featuring and rate of pulmonary embolism in COVID-positive patients

	**No VAX = 31**	**VAX = 26**	***P***
**Age**	63 ± 12	74 ± 14	< 0.052
**Charlson Comorbidity Index**	2	3	< 0.0002
**Pulmonary embolism-overall**	16	3	*p* 0.0064
**Without pulmonary embolism**	15	23

## Discussion

We observed a strong association between vaccination and PE in hospitalized COVID-19 patients: odds ratio for PE was significantly higher in not vaccinated patients, despite the exclusion of cases with concomitant main risk factors for VTE (trauma/surgery, thrombophilia, active cancer, bacterial sepsis) in order to minimize possible confounders. Age and comorbidities (Charlson Comorbidity Index) [4] were significantly more represented in vaccinated people, while unvaccinated patients had a more severe CT imaging. COVID-19 coagulopathy is a particularly complex manifestation. Endothelial cells, von Willebrand/ADAMTS13 axis, coagulation factors, fibrinolytic system, immunothrombosis, inflammatory cytokines, and anti-phospholipid antibodies are all involved in its determination [5]. We can just hypothesize a possible protective role of vaccination through an action on the immune system, modulating the inflammation-coagulation interface [6]. The hypothesis of a possible protective role of vaccination in preventing PE in hospitalized COVID-19 patients has not been explored. There are other studies in the literature that evaluate the incidence of thromboembolic events in vaccinated patients suffering from COVID-19 pneumonia, to evaluate the prognosis of these patients [7], or to evaluate the safety and efficacy of the vaccines used [8]. A retrospective population-based study on ambulatory patients showed a reduced venous thromboembolism incidence in vaccinated patients at 30-day follow-up [2]. According to a study in an emergency ward setting, unvaccinated patients had a 2.75-fold increased risk of COVID-associated PE during the Delta and Omicron periods compared to vaccinated or recovered patients [9]. The COVID-19 vaccine has been exhaustively demonstrated to be highly effective in terms of clinical benefit (reduced morbidity and mortality; reduced hospital duration). Nevertheless, because of rare/very rare hematological (idiopathic thrombotic thrombocytopenia, thrombotic thrombocytopenic purpura) and, above all, thrombotic complications (vaccine-induced thrombotic thrombocytopenia), this absolute benefit is not so clearly and widely accepted in term of public opinion [10, 11]. Our observation reinforces the effectiveness and safety of vaccination, opening to a possible protection from PE, one of the most feared complications of COVID-19. Of course, this is a single-center retrospective observational report. Nevertheless, our reported overall/ICU/Medical Ward PE prevalence data are in line with the current literature. The issue of the protective role of vaccination in COVID-19-associated VTE should be addressed in adequately designed and powered future studies: a population study in the city of Bologna is underway.

## Data Availability

The datasets used and/or analyzed during the current study are available from the corresponding author on reasonable request.
